# Perceived Causes and Attitudes Regarding Overindebtedness and Their Effects on Public Agreement With Government Financial Aid

**DOI:** 10.3389/fpsyg.2021.591765

**Published:** 2021-06-17

**Authors:** Jerônimo C. Soro, Mário B. Ferreira, Filipa de Almeida, Carla Sofia Silva, Joana Reis

**Affiliations:** ^1^CICPSI – Centro de Investigação em Ciência Psicológica, Faculdade de Psicologia, Universidade de Lisboa, Lisbon, Portugal; ^2^Faculdade de Psicologia, Universidade de Lisboa, Lisbon, Portugal; ^3^Universidade Católica Portuguesa, Católica Lisbon School of Business and Economics, Lisbon, Portugal

**Keywords:** overindebtedness, attitudes, causal attribution, beliefs, government support

## Abstract

In order to better understand how the problem of overindebtedness is perceived from a laypeople standpoint, Study 1 inquired both overindebted and non-overindebted consumers on the perceived causes of and attitudes toward the overindebted. Situational and dispositional factors were perceived to have similar impact as causes of overindebtedness, but non-overindebted consumers showed stronger agreement with those causes than overindebted consumers. Regarding attitudes, non-overindebted consumers tended to blame overindebted people for their situation rather than perceiving them as victims, whereas overindebted consumers showed the opposite pattern. Study 2 used a sample of (non-overindebted) consumers to assess the impact of perceived causes of overindebtedness, attitudes toward the overindebted, and political orientation on public support of government policies for aiding overindebted people. We discuss the contributions of the present findings to design public policies aimed at aiding overindebted households that are more aligned with the beliefs and attitudes of the general public.

## Introduction

Household debt levels in Western societies have seen a steady increase in the last few decades (e.g., Brown et al., 2005; Kida, 2009). Becoming indebted, by means of credit acquisition, has become a socially acceptable way of smoothing financial difficulties and enhancing the quality of life of households who have good reasons to believe they can afford to repay their debts with future income. From a macroeconomic perspective, consumer indebtedness has been considered to contribute to economic growth (Dickerson, 2008). The provision of credit is thus beneficial at an individual level and to society as a whole, provided that the ratio between consumers’ income and loan repayments allows consumers to fulfill debt services requirements.

However, the estimation of what is a financially manageable level of debt for a consumer, given her prospective income stream, is sometimes a complex matter due to both endogenous and exogenous risk factors. As a result, overindebtedness is bound to be a persistent phenomenon in the current consumer society. To illustrate, in Europe alone, between 2010 and 2018, an average of 11.1% of households were reported to be in arrears with some sort of credit (mortgage, utility bills, or hire purchase–Eurostat, 2020).

Although the causes of overindebtedness are multiple (e.g., Berthoud and Kempson, 1992; Van Staveren, 2001), evidence-based research reports (Dickerson, 2008, 2009; Disney et al., 2008; Russell et al., 2011; D’Alessio and Iezzi, 2013), converge on the observation that poverty and lack of resources play a key role in accumulating too much debt. As a result, proposed solutions to fight overindebtedness tend to include anti-poverty measures (e.g., social welfare, employment support) along with strategies geared toward dealing with other related aspects such as poor financial decision making and financial illiteracy.

However, the success of any public policy put forward to help overindebted households is likely to depend, to a substantial extent, on public approval. Although people’s attitudes toward poverty and beliefs concerning the causal attributions of poverty (and related governmental support) have already been explored elsewhere (Bullock, 1999; Bullock et al., 2003), surprisingly little research has considered people’s beliefs about the causes of overindebtedness and attitudes toward the overindebted. The main goal of the current research is to shed some light on these issues by surveying the perceived causes of overindebtedness (i.e., which risk factors are perceived to be more and less important), and attitudes toward the overindebted as well as public adherence to political measures to financially support overindebted consumers.

Furthermore, the social perception and judgment of consumers who have never been overindebted may differ from those who have firsthand experience with such extreme types of financial difficulties. To explore these differences, we also compare non-overindebted and overindebted consumers in terms of the perceived causes and attitudes regarding overindebtedness. Our findings contribute to better understand how the problem of overindebtedness is perceived from a laypeople standpoint while shedding light on how people may react to public policies and interventions aimed at attenuating the toll of overindebtedness.

Notwithstanding the different technical definitions of overindebtedness (e.g., Davydoff et al., 2008; Russell et al., 2011), there is a general consensus which regards a household as overindebted when its net resources (income and realizable assets) render it persistently unable to meet essential living expenses and debt repayments as they fall due (Stamp, 2009). In other words, overindebtedness may be defined as the persistent difficulty, or impossibility, of a household to pay its bills or debts.

Overindebtedness has considerable consequences both for individuals and for society, with households reporting reduced standard of living, deterioration of well-being, health, and financial exclusion (i.e., limited access to bank and credit services). Moreover, high levels of debt-per-income ratio are found to affect subsequent periods of recession and financial crisis, producing significantly larger contractions in economic activity and creating a feedback loop in which indebted households cut back in consumption, decreasing demand for products, which, in turn, decreases production and demand for workers, consequently increasing unemployment (Alleweldt et al., 2013).

Overindebtedness has both endogenous and exogenous factors as causes (Disney et al., 2008). Endogenous factors include financial imprudence frequently related to low levels of financial literacy coupled with poor budgeting skills (e.g., Lusardi, 2012; Lusardi and Scheresberg, 2013; Lusardi and Tufano, 2015). Impulsive consumerism and lack of self-control have also been associated to excessive levels of accumulated debt, with impulsive consumers showing more hyperbolic discounting and lower tolerance to delayed gratification (Vohs and Heatherton, 2000; Vohs and Faber, 2007; Gathergood, 2012). This relationship between impulsivity and over-indebtedness was recently confirmed in a meta-analysis conducted by Frigerio et al. (2020). Specifically, impulsivity was more strongly associated with unmanageable debt (i.e., overindebtedness) compared to (manageable) debt holding. Exogenous factors include unforeseen income shocks due to adverse life events (e.g., family breakdown) and macro-economic shocks (Alleweldt et al., 2013). A case in point is the 2010 sovereign debt crisis. This crisis led to an abrupt increase in taxation, unemployment, and severe income cuts (mostly in Southern European countries) that launched many households from the middle-class working sectors into extreme financial arrears and overindebtedness.

These two sources of indebtedness have led some researchers (e.g., Anderloni and Vandone, 2011) to suggest both “passive” and “active” types of overindebtedness. In passive overindebtedness, financial difficulties are the result of exogenous factors beyond an individual’s control, such as illness, job loss, or changes in macroeconomic variables. Active overindebtedness, on the other hand, is the result of borrowing more than one can pay back, due to factors such as individuals’ impulsivity, financial illiteracy, lax attitudes toward spending, among other personality dimensions and lifestyle behaviors (Lunt and Livingstone, 1991; Livingstone and Lunt, 1992; Pinto et al., 2000).

Households’ financial well-being is likely to depend on the interplay of several of these more endogenous (active) and exogenous (passive) factors as different combinations of such factors may lead to different profiles of indebtedness (Ferreira et al., 2020). Nevertheless, several sources of empirical evidence from European countries (Disney et al., 2008; Russell et al., 2011; D’Alessio and Iezzi, 2013) show that there is a strong connection between poverty and overindebtedness. Therefore, policies similar to those used to attenuate poverty are likely to be good candidates to address the social problem of overindebtedness.

People’s acceptance of measures to tackle poverty (such as welfare Government intervention) is related to lay theories of the causes of poverty (for a review, see Van Oorschot and Halman, 2000). Individuals’ attitudes toward the poor also appear to be related to the causal attribution of poverty and to the predisposition to help poor people (Cozzarelli et al., 2001; Tagler and Cozzarelli, 2013). However, overindebtedness and poverty are different social problems (even if they sometimes overlap) that may be differently perceived by the public. Indeed, while poverty is associated with low income and a lower socioeconomic status, overindebtedness results from the rise of a negative imbalance in the ratio between income and loan repayments, and thus may affect consumers from all socioeconomic strata. It is thus important to better understand people’s causal attributions of overindebtedness and attitudes toward the overindebted since interventions to tackle the rising levels of overindebtedness in society may depend, to a large extent, on the public support they receive.

Prior research on the perceived causes of overindebtedness, and on attitudes toward the overindebted is scarce. Lunt and Livingstone (1991) explored lay theories for personal debt using a network approach. They identified internal and external distal causes of debt (i.e., variables that are present in the network mostly or uniquely as causes and not consequences). This research, however, did not assess intensity or preference for each cause, so it was not clear whether people perceive some causes as more relevant than others. Despite extensive prior research on attitudes toward debt (e.g., Dessart and Kuylen, 1986; Livingstone and Lunt, 1992; Lea et al., 1993, 1995; Pinto et al., 2004), to the best of our knowledge there are no studies specifically focusing on people’s attitudes and beliefs toward the overindebted.

The two studies here reported contribute to overcoming this relative lack of research on attitudes and perceived causes of overindebtedness.

Study 1 investigated consumers’ perceived causes of overindebtedness and their attitudes toward the overindebted. The participants in this study were overindebted and non-overindebted consumers. Study 2 assessed consumers’ agreement with Government policies to financially support overindebted households. The proposed policies either involve direct financial support from the Government or the Government enforcing private creditors to suspend the payment of monthly installments, until overindebted consumers recover a financial balance. Additionally, participants in this study evaluated the extent to which these financial aid policies should be applied to all overindebted households or whether they should depend on the fulfilling of certain conditions (e.g., overindebted individuals would have to enroll in financial literacy courses in order to be covered by such Government policies). Study 2 further assessed the impact of perceived causes of overindebtedness, attitudes toward the overindebted, and political orientation in consumers’ public support of the aforementioned public policies.

In sum, Study 1 focused on the differences between overindebted and non-overindebted people regarding the measures of causal attribution, and attitudes toward the overindebted. Study 2 explored how these measures relate to political orientation and the willingness to accept proposed government policies to help overindebted consumers.

## Study 1

Given the partial overlap between overindebtedness and poverty, and since studies focusing on laypeople’s causal attributions of overindebtedness or attitudes toward the overindebted are scarce, in this first study we extrapolate data from the literature on poverty to overindebtedness to explore whether results from prior research on poverty involving these variables are generalizable to the case of overindebtedness.

Prior research indicates that middle income people attribute poverty more to individual rather than situational characteristics, while low-income people and welfare recipients tend to make more external than internal causal attributions (Feagin, 1975; Bullock, 1999).

Study 1 explores the extent to which non-overindebted consumers, when compared to their overindebted counterparts, also attribute overindebtedness more to individualistic rather than situational characteristics.

Past literature, using simple evaluative measures (i.e., *good* vs. *bad*) has revealed favorable attitudes toward impoverished people (Cozzarelli et al., 2001; Tagler and Cozzarelli, 2013). However, other studies of attitudes toward poverty and welfare point to a more complex results pattern (e.g., Feagin, 1972, 1975; Price et al., 1988; Smith and Stone, 1989; Lichter and Crowley, 2002; Hall et al., 2014). Frequently, attitudes toward the impoverished appear to reflect individualistic and situational explanations for poverty. The individualistic explanation emphasizes personal deficits as the primary cause of poverty, while the situational explanation highlights structural deficiencies within the economy (DiNitto, 2000; Mullaly, 2007), or uncontrollable factors such as personal misfortune or disability (e.g., Feagin, 1972, 1975; Cryns, 1977; Golding and Middleton, 1982). Similarly, attitudes toward impoverished people have been shown to involve blaming them for their financial situation (Lichter and Crowley, 2002; Park et al., 2007) or to perceiving them more as victims of socioeconomic dynamics (Clery et al., 2013).

In Study 1, we expect to find a similar pattern of results, that is, that non-overindebted participants, when compared to overindebted participants, show a tendency to blame more overindebted consumers for their difficult social economic circumstances.

### Materials and Methods

#### Participants

Three hundred and sixty-five overindebted (OI) and non-overindebted (NOI) participants took part in this study. The OI participants were consumers who sought financial assistance and counseling with an NGO for consumer defense (DECO–Portuguese Association for Consumer Defense) throughout 2017. The NOI participants were a convenience sample obtained from different public settings. Six participants from the latter sample identified themselves as OI and were coded as such in the analysis. The overall sample included 236 OI and 129 NOI participants.

#### Materials

The measure of causal attributions of overindebtedness was composed of 25 causes (risk factors) of overindebtedness, based on interviews with an independent sample of OI consumers. The measure of attitudes toward the overindebted was composed of eight items from the attitudes toward poverty scale of Atherton et al. (1993). The content of these items was adjusted by replacing “poor” and “poverty” with “overindebted” and “overindebtedness.” Two additional items were included, one related to passive overindebtedness (“Overindebted people have not had the same opportunities in life as other people”) and the other related to active overindebtedness (“Overindebtedness problems result from irresponsible spending from consumers”). The two measures were part of a larger survey which included other measures (for other research purposes) not described here.

#### Design and Procedure

The majority of OI participants (219) responded to the questionnaire in a paper format (those who responded on DECO premises) while a small number (17) responded by means of an editable computer file sent to them by e-mail (those who contacted DECO through their website or e-mail). NOI participants responded to the questionnaire only in a paper format. Participants also responded to socio-demographic questions (marital status, level of education, professional status, number of people in the household) and questions concerning financial aspects of their life (amount of debt and value of monthly installments for their credits, the total income of the household, and the total expenses of the household). Socio-demographic information of the sample is displayed in Table 1^1^.

**TABLE 1:** Socio-demographic characteristics of overindebted and non-overindebted samples of participants in Study 1.

	Overindebted	Non-overindebted
**Age**		
M (SD)	52.30 (11.66)	48.93 (17.61)
Valid N	86	128
Income (monthly)**		
M (SD)	1100.65 (562.54)	2103.50 (2176.07)
Valid N	154	120
Income (monthly) per capita **		
M (SD)	597.87 (372.34)	1042.84 (825.00)
Valid N	147	119
Debt**		
M (SD)	733.88 (944.51)	170.26 (222.74)
Valid N	149	113
Debt to income ratio*		
M (SD)	0.83 (1.89)	0.20 (0.17)
Valid N	140	55
Debt + expenses to income ratio*		
M (SD)	1.61 (2.19)	0.81 (0.50)
Valid N	83	55
People in the household*		
M (SD)	2.10 (1.00)	2.40 (1.27)
Valid N	152	126
**Education level**		
1st cycle (6–9 years old)	20 (12.82%)	3 (2.34%)
2nd cycle (10–11 years old)	13 (8.33%)	8 (6.25%)
3rd cycle (12–14 years old)	35 (22.43%)	26 (20.31%)
Secondary and Vocational ed. (15–17 years old	63 (40.38%)	42 (32.81%)
Higher education	25 (16.03%)	49 (38.28%)
Valid N	156	128
**Marital status**		
Single	37 (23.56%)	31 (24.21%)
Divorced/Separated	45 (28.66%)	23 (17.97%)
Married/Domestic partnership	64 (40.76%)	54 (42.18%)
Widowed	11 (7%)	20 (15.62%)
Valid N	157	128
**Professional status**		
Unemployed	31 (19.87%)	18 (14.4%)
Informal jobs	3 (1.92%)	7 (5.6%)
Retired	34 (21.79%)	41 (32.8%)
(Self-)Employed	88 (56.41%)	59 (47.20%)
Valid N	156	125

*p < 0.05, **p < 0.001.

For the measure of causal attribution, participants were asked to evaluate how much each potential cause contributes to creating a situation of overindebtedness using a 5-point scale (1—*Does not contribute at all*, 5—*Contributes very much*). For the measure of attitudes toward the overindebted, participants were asked to express their agreement with each presented item on a 5-point scale (1–*Completely disagree*, 5–*Completely agree*). Only the participants who responded to all the questions in each of the measures (valid data) were considered for data analyses.

### Results and Discussion

#### Principal Components Analysis

Data from each measure was subjected to principal components analyses (PCA) with VARIMAX rotation.

Preceding the PCA, a descriptive analysis of the items of each measure was performed to obtain information about the symmetry of the distribution of the items. In both measures and for some items, the ratio of skewness to standard error of skewness was above the | 2| range (e.g., “Unemployment” in the measure of causal attributions of overindebtedness; “Overindebted people are less capable in general compared to other people” in the measure of attitudes toward the overindebted). However, the absolute values of *skewness* for all items of both measures were lower than 3, which can be considered non-problematic in terms of distribution (Kline, 2011). Therefore, for each measure, all items were included in the subsequent PCA. Items with loadings below 0.5 or with loadings above 0.4 in more than one component were eliminated (see Supplementary Tables 1, 2).

Regarding the causal attribution of overindebtedness with valid data from 250 participants (136 OI and 114 NOI), four dimensions were obtained accounting for 64.81% of response variability (see Table 2). The first dimension was named “situational factors” (eigenvalue of 11.39, α = 0.92). It includes societal causes of a personal (e.g., divorce/separation) and professional nature (e.g., unemployment), corresponding to a passive type of overindebtedness. The second dimension was named “dispositional factors” (eigenvalue of 1.96, α = 0.88). It refers to causes such as consumption impulsiveness, financial illiteracy, among other related aspects, indicating an active type of overindebtedness. Two remaining dimensions, both composed of only two items and with lower eigen values (<1.67), were not further considered in the following analysis.

**TABLE 2:** Principal components analysis of the causal attribution questionnaire presenting items loadings on each dimension.

Items	1	2	3	4
1. Increase of the household	0.660			
2. Late salary payments	0.816			
3. Unstable work conditions	0.756			
4. Salary cuts	0.718			
5. Unemployment	0.826			
6. Unemployment of spouse	0.803			
7. Divorce/separation *	0.592			
8. Disease/work incapacitation *	0.560			
18. Failure in individual business ventures *	0.549			
21. Victim of scam or fraud	0.614			
9. Impulsive buying		0.685		
10. Bad management of monthly budget		0.731		
11. Lack of appropriate knowledge about credit		0.605		
15. Easy access to credit		0.748		
20. Excessive resort to credit (Credit cards, personal credit)		0.875		
25. Debt “Snowball” effect		0.687		
12. Misfortune in financial issues			0.784	
13. Problems with Guarantor			0.614	
23. Difficulty adjusting to the new economic reality				0.673
24. Current financial crisis				0.864

*Items not included in the questionnaire used in Study 2.

In the measure of attitudes toward the overindebted, with the valid data from 286 participants (164 OI and 122 NOI), three dimensions emerged accounting for 50.99% of the response variability (Table 3). The first was named “victimizing”^2^ (eigenvalue of 2.11, α = 0.61), comprising beliefs of overindebted people as victims of the circumstances and deserving of help. The second dimension was named “blaming” (eigenvalue of 1.9, α = 0.53) and it refers to beliefs that the overindebted are to blame for their financial condition. A third dimension composed of only two items and with an eigenvalue close to one (1.09) was not further considered in the following analysis.

**TABLE 3:** Principal components analysis of the attitudes toward the overindebted questionnaire presenting items loadings on each dimension.

Items	1	2	3
1. Overindebted people have not had the same opportunities as other people.	0.660		
2. People are overindebted usually due to circumstances beyond their control.	0.505		
5. Overindebted people are discriminated against.	0.723		
7. Overindebted people should not be blamed for their misfortune.	0.636		
4. If overindebted people worked harder, they could escape their debt situation.0		0.782	
6. Unemployed overindebted people could find jobs if they tried harder.		0.704	
10. Overindebted situations are due to overindebted people’s irresponsible spending.		0.518	
8. Overindebted people are less capable, in general, compared to other people.			0.782
9. Overindebted people have a different set of values to other people.			0.806

#### Comparisons Between Overindebted and Non-overindebted Participants

Next, we tested for differences between OI and NOI participants in the PCA dimensions that emerged from each measure (causal attribution of overindebtedness and attitudes toward the overindebted). To control for the potential confounding effects of education and household income, level of education and household income per capita (i.e., income divided by the number of household members) were included as covariates in all the analyses.

##### Causal attribution

The analysis was performed with valid data from 244 participants (127 OI and 117 NOI). Both causal attribution dimensions were considered to contribute to overindebtedness, with mean values above the mid-point of the scale.

**FIGURE 1:**
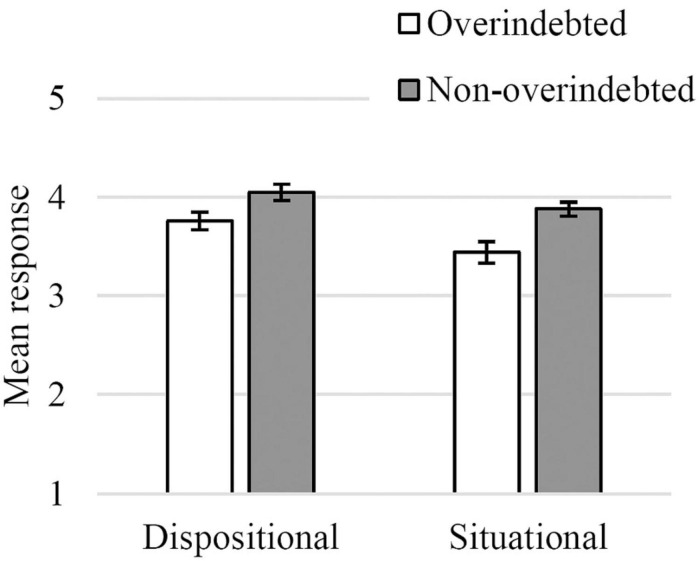
Causal attribution for each dimension in both groups of participants (1—*Does not contribute at all*, 5—*Contributes very much*; bars indicate standard error).

A 2 X 2 repeated measures ANCOVA was computed with causal attribution (situational factors, dispositional factors) as a within-participants variable, group (OI, NOI) as a between-participants variable, and household income and education level as covariates. The ANCOVA yielded a main effect of group [*F*_(1, 240)_ = 6.63, *p* = 0.011, η*_*p*_*^2^ = 0.03], such that NOI participants showed greater causal attribution scores (*M* = 4.06, *SE* = 0.17) than OI participants (*M* = 3.74, *SE* = 0.02). There was also an interaction between causal attribution and household income, [*F*_(1, 240)_ = 4.62, *p* = 033, η*_*p*_*^2^ = 0.02], such that higher income was associated with higher dispositional attributions (β = 0.132, *p* = 0.05) but had no effect on situational attributions. No other effects were significant (Figure 1 and Suplementary Table 3).

In short, NOI participants made stronger attributions overall than OI participants. Furthermore, wealthier households made stronger dispositional (but not situational) attributions of overindebtedness. Contrary to our expectations, non-overindebted consumers, when compared to their overindebted counterparts, did not attribute overindebtedness more to dispositional than to situational characteristics.

##### Attitudes toward the overindebted

The analysis was performed with valid data from 223 participants (104 OI and 119 NOI). Both victimizing and blaming attitudes toward the overindebted were close to the mid-point of the scale, indicating moderate attitudes.

**FIGURE 2:**
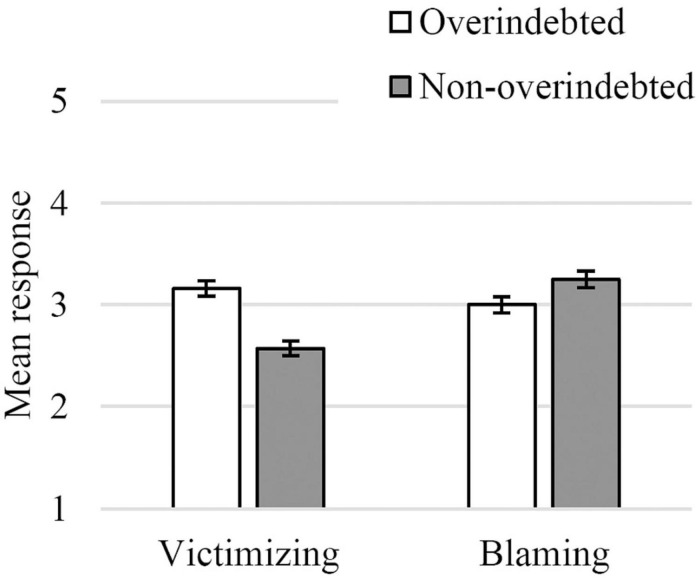
Attitudes toward the overindebted for each dimension in both groups of participants (1–*Completely disagree*, 5–*Completely agree*; bars indicate standard error).

A 2 X 2 repeated measures ANCOVA was computed with attitudes (victimizing, blaming) as a within-participants variable, group (OI, NOI) as a between-participants variable, and household income and education level as covariates. The ANCOVA revealed a main effect of attitudes, [*F*_(1, 219)_ = 9.90, *p* = 0.002, η*_*p*_*^2^ = 0.04], such that blaming attitudes toward the overindebted (*M* = 2.69, *SE* = 0.12) were higher than victimizing attitudes (*M* = 2.42, *SE* = 0.12). There was also an interaction between attitudes and group, [*F*_(1, 219)_ = 24.16, *p* < 0.001, η*_*p*_*^2^ = 0.10], such that the OI participants tended to have more victimizing attitudes toward overindebted people (*M* = 3.16, *SE* = 0.08) than NOI participants (*M* = 2.57, *SE* = 0.07), [*F*_(1, 219)_ = 23.03, *p* < 0.001], whereas NOI participants revealed more blaming attitudes toward overindebted people (*M* = 3.25, *SE* = 0.08) when compared to OI participants (*M* = 3.00, *SE* = 0.09; Figure 2).

There was also a main effect of level of education, [*F*_(1, 219)_ = 4.13, *p* = 0.043, η*_*p*_*^2^ = 0.02] and an interaction between attitudes and both household income, [*F*_(1, 219)_ = 5.71, *p* = 0.018, η*_*p*_*^2^ = 0.03], and education level, [*F*_(1, 219)_ = 12.02, *p* < 0.001, η*_*p*_*^2^ = 0.05]. Higher education levels were associated with less blaming (β = −0.25, *p* < 0.001) but did not affect victimizing (β = 0.07, *p* = 0.282). Higher household income was marginally associated with more blaming attitudes (β = 0.13, *p* = 0.060), but did not affect victimizing attitudes toward overindebted people (β = −0.10, *p* = 0.138).

As expected, NOI participants not only expressed more blaming attitudes toward overindebted people but also showed a greater tendency to refuse to treat them as victims of a lack of opportunities and misfortune. Also, more educated consumers showed less blaming attitudes, whereas wealthier participants tended to blame overindebted people more for their financial situation.

By exploring how overindebtedness is perceived from a laypeople standpoint, Study 1 points to relevant differences in the attitudes toward overindebted people of OI and NOI participants. One important question is how these attitudes influence people’s support for welfare policies of resource re-distribution aimed at helping overindebted households.

## Study 2

Study 2 used the same measures of perceived causes and attitudes toward overindebtedness of Study 1. Furthermore, participants’ agreement with Government policies to financially support overindebted people was also assessed. These policies either involved (a) direct financial support from the Government (i.e., lending money at zero interest rates to overindebted people to pay their debts); or (b) indirect support on the part of the Government by enforcing private creditors to suspend the payment of monthly installments (with no interest accrual) until consumers recover from their situation of overindebtedness. In addition, participants were inquired on the extent to which they believed that this financial aid should depend on certain conditions (e.g., the overindebted would have to enroll in financial literacy courses) or should be unconditional.

Finally, since prior research concerning the perceived causes of poverty and support of welfare policies revealed marked differences between left and right-wing individuals (e.g., Furnham, 1982) and given the connections between poverty and overindebtedness, Study 2 also assessed participants’ political orientation. Based on research in the domain of poverty, participants with a more right-wing political orientation (when compared to more left-wing participants) were expected to (a) perceive dispositional causes as more important than situational causes of overindebtedness; (b) display more blaming attitudes and less victimizing attitudes toward the overindebted; (c) be less supportive of Government financial aid (particularly when involving financial losses for private credit institutions); and (d) be more prone to imposing conditions for overindebted consumers to obtain financial aid.

In short, the main goals of Study 2 were to (a) assess public support of welfare policies aimed at helping overindebted consumers; and (b) explore the mediating role of perceived causes of overindebtedness and attitudes toward the overindebted in the relation between political orientation and people’s support of welfare policies.

### Materials and Methods

#### Participants

One hundred and ninety-nine participants were recruited via Prolific and completed the survey online. Three participants were removed from the analysis. Two for responding incorrectly to the attention check question and one for responding to the entire survey in less than 4 min, which was deemed insufficient time for attentive completion of the survey. The remaining 196 participants included 104 males and 92 females with a mean age of 38.80 (*SE* = 7.17). Further demographic characterization of the participants is presented in Table 4.

**TABLE 4:** Socio demographic information of the sample in Study 2.

Variable	M (SD)
Income (monthly)	1832.57 (1110.44)
N° of people in the household	2.59 (1.14)
Household average income	808.172 (599.44)
Monthly value paid for credit installments	376.88 (469.59)
Monthly expenses (without credit)	788.72 (507.68)
Debt to income ratio	0.22 (0.22)
Debt+expenses to income ratio	0.74 (0.64)
Political orientation	4.45 (1.42)
**Education Level**	**N (% of total)**
2nd cycle (10–11 years old)	2 (1.02%)
3rd cycle (12–14 years old)	2 (1.02%)
Secondary and Vocational ed. (15–17 years old)	60 (30.61%)
Higher education	132 (65.31%)
**Professional status**	
Unemployed	8 (4.08%)
Informal jobs	4 (2.04%)
Retired	2 (1.02%)
(Self-)Employed	175 (89%)
Student	1 (0.51%)
Other	6 (3.06%)

#### Materials

The same measurement tools of Study 1 were used to measure causal attributions and attitudes toward the overindebted. The causal attribution measure was reduced to be slightly more succinct. To create this shortened version, items with loadings below 0.60 were removed.

Two different versions of a governmental measure to financially support overindebted consumers were created. One involved direct financial support from the Government: “The government should help overindebted people by lending them money (without interest) for them to pay their monthly installments to their creditors until they recover from their situation of overindebtedness.” In the second version the Government financial support was indirect, by suspending the debt service (monthly installments including interest) of overindebted consumers: “The government should help overindebted people by enforcing creditors to suspend the charging of monthly installments (without an interest accrual) until they recover from their situation of overindebtedness.”

Four additional questions were developed to evaluate the extent to which participants would consider that the Government’s measure of financial support should be unconditional or should be contingent on (a) prohibiting overindebted consumers from incurring more debt over the next 10 years; (b) the obligatory enrolment of overindebted consumers in courses on financial literacy; (c) cases of overindebtedness resulting from unpredictable causes (e.g., death in the family); (d) cases of overindebtedness among consumers of a low socio-economic status.

The first of these conditions taps into the belief that overindebtedness stems from impulsive consumer behavior and a lack of self-control. The second condition is based on the underlying notion that overindebtedness is the result of financial illiteracy. The third and fourth conditions stress the importance of unpredictable or systemic external causes of overindebtedness as a requirement to obtain Government financial support.

#### Design and Procedure

Participants began by responding to socio-demographic questions (age, sex, level of education, professional situation, and household size) as well as questions on their financial situation (income, expenses, credit installments, and savings). They then indicated their political orientation on a rating scale, from 1 (*Extreme left*) to 9 (*Extreme right*; Jost, 2006). Participants then responded to the items in causal attribution and attitudes questionnaires using the same rating scale (1–*Totally disagree*, 5 *Totally agree*). Following this, participants were randomly assigned to receive one of the two versions of the government measure to financially support overindebted consumers and expressed their agreement with the measures (using the same rating scale but with 7-points). Finally, all the participants were asked to imagine that the Government had decided to implement the presented financial support measure and to evaluate the extent to which they agreed that Government financial support should be applied unconditionally or whether it should only be applied under specific conditions (see materials). Participants responded to each of these items using a 7-point rating scale where “1” corresponded to the unconditional application of the financial support measure to all cases of overindebtedness and “7” corresponded to full agreement with the presented condition for the application of government financial support.

### Results and Discussion

#### Causal Attribution

A Generalized Linear Model (GLM) with causal attribution (situational factors, dispositional factors) as a within-participants factor and political orientation as a continuous factor yielded only a significant main effect of causal attributions, [*F*_(1, 194)_ = 8.63, *p* = 0.004, η*_*p*_*^2^ = 0.04]. Participants considered situational factors (*M* = 3.77, *SE* = 0.12) to contribute less than dispositional factors (*M* = 4.31, *SE* = 0.12) to overindebtedness, regardless of their political orientation.

#### Attitudes Toward the Overindebted

A GLM with attitude (victimizing, blaming) as a within-participants factor and political orientation as a continuous factor yielded only an interaction between political orientation and attitudes, [*F*_(1, 194)_ = 4.90, *p* = 0.028, η*_*p*_*^2^ = 0.03]. While a more right-wing political orientation was associated with more blaming (β = 0.18, *p* = 0.014), political orientation had no effect on victimizing (β = −0.08, *p* = 0.253, Supplementary Table 4).

#### Perception of Support Measures for Overindebted Participants

A GLM with type of support measure (Government direct support and Government indirect support) as a between-participants factor and political orientation as a continuous factor yielded only a significant effect of type of support measure, [*F*_(1, 192)_ = 4.22, *p* = 0.041, η*_*p*_*^2^ = 0.02], in which agreement with Government indirect support was higher (*M* = 4.15, *SE* = 0.17) than agreement with direct support (*M* = 3.85, *SE* = 17).

#### Agreement With Conditions for Support Measures

**FIGURE 3:**
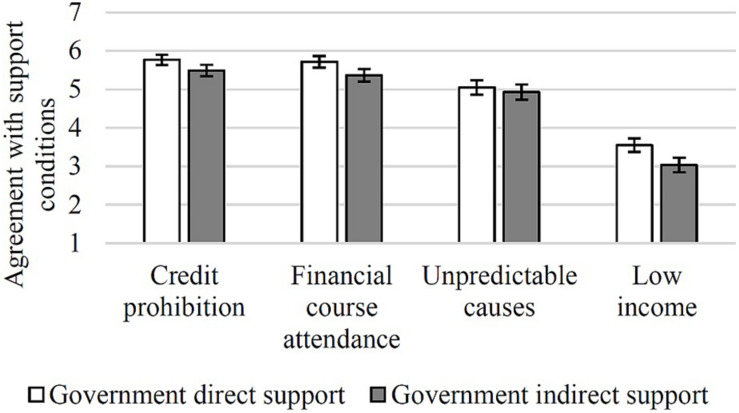
Participants agreement with conditions for overindebted people to receive financial aid for both types of support measure (1—*Agreement with unconditional support*, 7—*Agreement with conditional support*; bars represent standard error).

A GLM was computed with type of support measure (Government direct support and Government indirect support) as a between-participants factor, conditions for support measures (credit prohibition, financial course attendance, unpredictable causes, low income) as a within-participants factor, political orientation as a continuous factor and agreement with the conditions for support measure as the dependent variable. The GLM yielded a main effect of the type of support measure, [*F*_(1, 192)_ = 4.48, *p* = 0.036, η*_*p*_*^2^ = 0.02], with higher overall agreement with the imposition of conditions for Government direct support (*M* = 5.02, *SE* = 0.10 than for Government indirect support (*M* = 4.70, *SE* = 0.10). A main effect of condition was also observed, [*F*_(3, 576)_ = 9.03, *p* < 0.001, η*_*p*_*^2^ = 0.04]. *Post-hoc* comparisons (with Bonferroni correction) showed higher agreement with both credit prohibition (*M* = 5.63, *SE* = 0.10) and financial course attendance (*M* = 5.54, *SE* = 0.11), followed by agreement with limiting financial aid to unpredictable causes of overindebtedness (*M* = 4.98, *SE* = 0.13), and by agreement with limiting financial aid to low-income households (*M* = 3.28, *SE* = 0.13; all *p*s < 0.001; see Figure 3).

In sum, there was a tendency to disagree more with the direct Government support measure when compared to the indirect support measure. In other words, participants appear to be less supportive of a direct use of taxpayers’ money to help overindebted people.

Additionally, participants were in favor of imposing conditions that the overindebted must fulfill before receiving Government financial support, particularly in the case of Governmental direct support.

More specifically, participants were (a) in favor of prohibiting overindebted people from contracting new debt; (b) in favor of mandatory financial literacy courses; and (c) in favor of limiting access to the financial support measures to cases of overindebtedness resulting from unpredictable causes. However, participants were against the attribution of financial support only to the overindebted of low socio-economic levels. This results pattern may suggest an inclination to perceive impulsive consumer behavior and lack of self-control, as well as financial illiteracy, as factors underlying overindebtedness. Furthermore, conditioning Government financial support to unpredictable causes of overindebtedness may indicate that those who become overindebted due to imprudent financial behavior are perceived as being less deserving of Government aid. Finally, by not limiting Government aid to cases of overindebtedness that overlap with low income, participants appear to concede that overindebtedness is a societal problem that affects consumers from different socio-economic levels (and not only poor people).

#### Mediation Model

**FIGURE 4:**
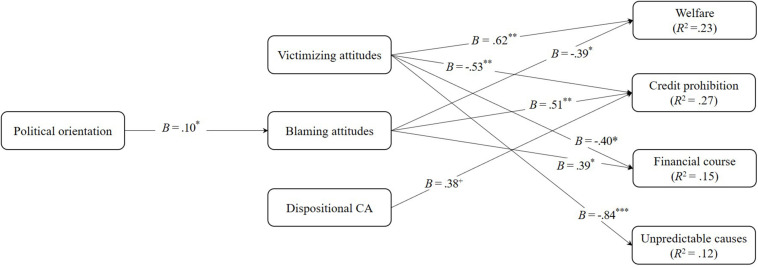
Model examining the mediating role of people’s attitudes toward the overindebted and perceived causes of overindebtedness in associations between political orientation and people’s agreement with financial support measures. For ease of interpretation, only significant effects are depicted. ^+^*p* < 0.10; *p < 0.05; **p < 0.01; ***p < 0.001.

To analyze the mediating role of perceived causes of overindebtedness and attitudes toward the overindebted in the impact of political orientation on participants’ agreement with the financial support measures (and their conditions), a multi-mediator path analysis model was tested, controlling for the version of governmental measure to financially support overindebted consumers (i.e., Government direct support vs. Government indirect support), household income, and participants’ level of education. This analysis was performed with the use of *MPlus* 7.2 (Muthén and Muthén, 1998-2012). Covariances between residuals among the mediators (Table 5) and among criterion variables (Table 6) were allowed in the model, of which only the significant covariances were retained. To test the indirect effects of political orientation on participants’ agreement with the support measures, through perceived causes of overindebtedness and attitudes toward the overindebted, bootstrap estimation was used with 5,000 subsamples to derive the 95% confidence interval for the indirect effects (Preacher and Selig, 2012). The following fit indexes and criteria were used as indicative of a good model fit: the comparative fit index (CFI) above 0.95, the root mean square error of approximation (RMSEA) and standardized root mean residual (SRMR) below 0.08 (Hu and Bentler, 1999; Kline, 2011). Results revealed a good model fit: [*χ*^2^(7) = 11.87, *p* = 0.105; CFI = 0.99; RMSEA = 0.06, 90% CI: 0.00, 0.12; SRMR = 0.02]. The model results are depicted in Figure 4.

**TABLE 5:** Covariances among residuals of the mediator variables.

Variable		1	2	3
1.	Victimizing attitudes	–		
2.	Blaming attitudes	−0.21**	–	
3.	Situational CA	0.19**	−0.10*	−
4.	Dispositional CA	*n.s.*	0.09*	0.11**

CA, Causal attributions; n.s., non-significant. *p < 0.01; **p < 0.001.

**TABLE 6:** Covariances among residuals of the criterion variables.

Variable		1	2	3	4
1.	Support measure	–			
2.	Credit prohibition	*n.s.*	–		
3.	Financial course	*n.s.*	0.63***	–	
4.	Unpredictable causes	*n.s.*	0.55**	*n.s.*	−
5.	Low income	−0.54***	*n.s.*	−0.32*	*n.s.*

n.s., non-significant. *p ≤ 0.05; **p < 0.01; ***p < 0.001.

As shown in Figure 4, the model results revealed a significant effect of political orientation only on blaming attitudes, *B* = 0.10, *SE* = 0.04, *p* = 0.019. More specifically, the more right-wing participants were, the higher their blaming attitudes.

Regarding the effects of attitudes toward the overindebted, the results showed significant effects of attitudes of victimizing on agreement with support measures *B* = 0.62, *SE* = 0.19, *p* = 0.001, and on the following conditions for obtaining financial support: credit prohibition, *B* = −0.53, *SE* = 0.17, *p* = 0.002, financial course attendance, *B* = −0.40, *SE* = −0.17, *p* = 0.021, and unpredictable causes, *B* = −0.84, *SE* = 0.22, *p* < 0.001. In other words, the stronger the participants’ victimizing attitudes toward overindebted people, the more they believed that the government should help those people, and the less they believed that such help should be contingent on prohibiting them from incurring additional debt for the following 10 years, on their obligation to attend a financial literacy course, and on the need for their overindebtedness having resulted from unpredictable causes.

Results also revealed significant effects of blaming attitudes on agreement with support measures, *B* = −0.39, *SE* = 0.15, *p* = 0.012, credit prohibition, *B* = 0.51, *SE* = 0.15, *p* < 0.001, and financial course attendance, *B* = 0.39, *SE* = 0.14, *p* = 0.006. In other words, the stronger the participants’ blaming attitudes toward indebted people, the less they believed that the government should help them, and the more they believed that such help, when given, should be contingent on credit prohibition for 10 years and the obligation to attend a financial literacy course.

Results also revealed significant indirect effects of political orientation, via blaming attitudes, on agreement with support measures −0.04, *SE* = 0.02, 95% CI [−0.087, −0.001], and two conditions for government financial support: (a) credit prohibition, *B* = 0.05, *SE* = 0.03, 95% CI [0.004, 0.107]; and (b) financial course attendance, *B* = 0.04, *SE* = 0.02, 95% CI [0.001, 0.009]. In other words, the more right-wing the participants were, the stronger their blaming attitudes toward overindebted people, and the stronger these attitudes, the more they believed that government financial support should be contingent on (a) prohibiting overindebted consumers from incurring more debt over the next 10 years, and (b) the obligation of overindebted consumers attending financial literacy courses.

There were no significant effects of participants’ causal attributions on their agreement with financial support measures.

Finally, theoretically expectable significant residual covariances among mediators (Table 5) and among criterion variables (Table 6) were also observed. Among the mediators, positive interrelations were found between the residuals for: (a) victimizing attitudes and situational attributions; (b) blaming attitudes and dispositional attributions; and (c) both dimensions of causal attributions. Conversely, negative interrelations were found between the residuals for (a) victimizing and blaming attitudes, and (b) situational attributions and blaming attitudes.

Fewer significant residual covariances were found among the criterion variables. More specifically, positive interrelations were found between the residuals for agreement with the financial support measure contingent on credit prohibition, financial course attendance, and unpredictable causes, while negative interrelations were found between the residuals for agreement with support measures and the support measure conditional on a (low) socio-economic status. A negative interrelation was also found between the residuals for financial support contingent on financial course attendance and low socio-economic status.

## General Discussion

This research investigated how the problem of overindebtedness is perceived from a laypeople standpoint. Study 1 explored the differences between overindebted (OI) and non-overindebted (NOI) consumers regarding the perceived causes of overindebtedness and attitudes toward overindebted people. Study 2 explored the impact of political orientation on these two measures, as well as the relationship between political orientation, perceived causes of overindebtedness, and attitudes toward the overindebted on the willingness to accept public policies to help overindebted consumers.

Given the scarcity of research on the perceived risk factors and causal attributions of overindebtedness, we briefly compare our results with findings from the literature on poverty. Causal attributions of poverty made by Portuguese consumers have been shown to be more socially situated, and less dispositional (Van Oorschot and Halman, 2000). In contrast, our findings suggest that Portuguese consumers consider dispositional factors of overindebtedness to be more important than situational factors (although this tendency was only statistically significant in Study 2). Also, in contrast with the research on the causal attribution of poverty, political orientation, assessed in Study 2, did not moderate causal attributions of overindebtedness.

Regarding attitudes toward the overindebted, and as expected, OI participants showed more victimizing than blaming attitudes whereas NOI participants showed the opposite pattern; they perceived the overindebted as more blameful for their financial problems and less as a victim of social circumstances beyond their control. Study 2 replicated these results for the NOI participants and found that political orientation interacted with attitudes toward the overindebted such that a more right-wing political orientation was associated with more blaming of overindebted consumers for their financial situation.

Study 2 also assessed public support of welfare policies aimed at helping overindebted consumers (involving either direct or indirect financial aid from the Government). In addition, participants were asked if these welfare policies should apply to all cases of overindebted households unconditionally or should be contingent on specific eligibility conditions.

Our findings show more agreement with the indirect compared to the direct Government support measure. Furthermore, when asked to consider concrete scenarios for the implementation of these welfare measures, participants were mostly in favor of imposing limiting conditions for receiving direct Government financial support. This may suggest that public opinion is less supportive of a direct use of taxpayers’ money to help overindebted people, and more prone to imposing limiting conditions for this support.

In terms of the specific conditions to benefit from Government financial support, consumers from Study 2 were in favor of prohibiting overindebted people from contracting new debt, which is in agreement with the higher causal attributions made for dispositional factors and may be seen as suggesting that public opinion perceives impulsive consumer behavior and lack of self-control as causes of overindebtedness. Participants were also in favor of imposing mandatory financial literacy courses, suggesting a tendency to associate overindebtedness with financial illiteracy. They also agreed with limiting financial aid to cases of overindebtedness that were due to unforeseen causes, indicating that those who become overindebted due to careless financial behavior might be perceived by the general public as being less deserving of Government financial aid measures. Lastly, participants tended to consider that all overindebted households, and not only overindebted households of low socio-economic levels should be eligible for Government financial support, which may indicate that overindebtedness is (accurately) perceived by the public as a societal problem that affects not only impoverished people but households from different socio-economic levels.

Study 2 further explored the mediating role that perceived causes of overindebtedness and attitudes toward the overindebted might have on the relationship between political orientation and support of (a) the proposed welfare policies; and (b) the presented welfare policies’ limiting conditions. Perceived causes of overindebtedness did not play a significant role in the mediation model. As for attitudes toward the overindebted, blaming attitudes mediated the effect of political orientation on the beliefs that government financial support should be contingent on (a) prohibiting overindebted consumers from incurring more debt, and (b) obligatory financial literacy courses for the overindebted. In other words, a more right-wing political orientation was associated with a greater blaming attitude toward the overindebted (i.e., a tendency to perceive the financial situation of the overindebted as the result of a lack of hard work and irresponsible spending); and, in turn, stronger blaming attitudes were associated with the two abovementioned conditions for the overindebted to obtain access to financial aid. This agrees with a worldview where the overindebted lack self-control (to work hard enough and/or inhibit impulsive consumption) and are financially illiterate (fostering irresponsible spending). Furthermore, these findings are aligned with prior research suggesting that right-wing political orientation is associated to beliefs that hierarchical differences between individuals legitimately reflect differences in effort and ability (Jost et al., 2003; Graham et al., 2009), whereas left-wing political orientation is associated to beliefs that the existing social differences stem mostly from situational factors such as luck (Kluegel and Smith, 1981; Jost et al., 2009). Such beliefs about the legitimacy of the social hierarchy’s naturally impact attitudes toward policies involving redistribution of wealth (Graham et al., 2009; Crawford et al., 2015).

Furthermore, and regardless of political orientation, stronger blaming attitudes were associated with less agreement with the proposed government financial aid measures for the overindebted. In contrast, stronger victimizing attitudes (i.e., perceiving overindebtedness as the result of a lack of opportunities and social circumstance beyond one’s control) were associated with more agreement with government financial aid measures, and were also associated with less agreement with making government aid contingent on (a) not incurring additional debt; (b) obligatory financial literacy courses; and (c) limiting it to cases of overindebtedness resulting from unpredictable causes.

In sum, blaming and victimizing attitudes appear to play important and opposite roles in the public support of welfare policies involving Government financial aid to overindebted individuals. Blaming attitudes lead to decreased endorsement of these policies and to imposing more conditions in order for one to be able to benefit from these policies, whereas victimizing attitudes were associated with greater endorsement of the same policies in a more unconditional manner.

More right-wing political orientation was associated with stronger blaming attitudes and with two conditions for Government financial support of the overindebted (credit prohibition and obligatory financial course attendance) indirectly via blaming attitudes. The specific version of the welfare policy (direct financial aid from the Government or indirect help by enforcing creditors to suspend the charging of monthly installments) did not play a significant role in the model.

### Limitations and Social Implications

The reported studies present some limitations. First, the measurement tools used to assess attributions and attitudes toward overindebtedness have not been validated for the Portuguese population. Second, the samples of consumers used were not representative of the population. Third, in Study 1, the percentage of consumers who did not fully respond to one or both of the measurement tools considerably reduced the valid sample (since only consumers who completed all questions in each of the measures were considered for statistical analyses). Fourth, several reported findings are correlational in nature, which makes it impossible to clearly establish causality. Future research involving the validation of the measurement tools with new independent samples of consumers and using longitudinal designs to establish causality, could provide the necessary conditions to overcome such limitations and to further test some of the preliminary conclusions and hypotheses raised herein.

Notwithstanding the preliminary nature of the present findings, the reported results not only provide some clues for a better understanding of how the problem of overindebtedness is perceived from a laypeople standpoint, but also shed some light on how people may react to public policies and interventions aimed at aiding overindebted households. These are key social issues as the success of any Government welfare measure in democratic societies is considerably dependent on public support (Brooks and Manza, 2006).

In this respect, our findings suggest that laypeople hold moderately positive opinions concerning welfare measures that involve the Government’s financial aid of the overindebted. Political orientation, although relevant, plays only a partial and relatively small role in people’s responses to the proposed welfare measures. This may be due to the fact that most participants described their political orientation as centrist (center-left or center-right), suggesting moderate political views, which may have limited the explanatory power of this factor.

In contrast, the prevalence of a blaming attitude toward the overindebted is associated with reduced public acceptance of Government financial aid and to increased support of limiting conditions for those benefiting from this aid. On the other hand, the prevalence of a victimizing attitude among the public is associated with the acceptance of Government financial aid and support of a more generalized coverage of these welfare interventions (covering a larger proportion of overindebted households). Thus, our findings may also contribute to anticipating the public acceptance of interventions that may be used to aid overindebted households in modern societies.

Public opinion and attitudes toward the overindebted are not fixed and may vary as a function of the perceived sources of the problem. To illustrate, after the bailout loan of Portuguese sovereign debt in 2010, the Portuguese society was besieged by severe austerity due to so-called punitive interest rates often justified as the consequence of years of collective overspending. Political accounts as such are likely to promote blaming attitudes toward those facing debt and financial difficulties with downstream negative consequences in terms of public acceptance of welfare measures (Brooks and Manza, 2006). In contrast, a large proportion of the population has now fallen victim to an economic lockdown due to the COVID 19 pandemic, which is launching the world into a new financial crisis. Economic threats as this one have been found to predict prosocial tendencies through empathic concerns (Alonso-Ferres et al., 2020). Victimizing attitudes are now more likely to prevail in the political discourse and public opinion, making it easier for governments to implement welfare measures that involve, in many cases, direct financial aid for those in financial difficulties similar to the support measures we used in Study 2.

Indeed, since the start of the pandemic that governments worldwide have been adopting financially supporting policies in an effort to attenuate a new worldwide financial crisis. This, by itself, might be shaping the public opinion leading to increased public support for these measures (Jordan, 2013). Future research should thus take this opportunity to better understand if and in what conditions governmental measures of financial support increase consumers trust and social solidarity toward indebted households. This is a relevant research goal since trust in the governmental system and solidarity toward the recipients of social welfare have been found to be important predictors of public support for welfare measures (Daniele and Geys, 2015). In contrast, the belief that others might use the welfare system inappropriately has been found to deteriorate interpersonal trust on welfare measures (Daniele and Geys, 2015). Perhaps these effects are strong enough as to occur above and beyond consumers political orientation.

The broader point we would like to make is that laypeople’s attitudes toward the overindebted are likely to be malleable to a certain extent, depending on social norms of meritocracy, trust and solidarity that may become more or less dominant as the result of major socio-economic events (as the economic shock stemming from the Covid 19 world pandemic) and on how these events are perceived and interpreted.

Our findings are among the first to shed light on a deeper understanding of the relationship between (blaming and victimizing) attitudes and the public’s reactions to Government welfare policies targeting overindebted consumers. They suggest that there is value for Governments in democratic societies to assess the degree to which the general public blames overindebted consumers for their situation or perceives them as victims, as this makes it possible to anticipate the support for public interventions, as well as to specify conditions to prevent and reduce overindebtedness.

## Data Availability Statement

The raw data supporting the conclusions of this article will be made available by the authors, without undue reservation.

## Ethics Statement

The studies involving human participants were reviewed and approved by the Ethics committee of the Faculty of Psychology, University of Lisbon. The participants provided their written informed consent to participate in this study.

## Author Contributions

JS, MF, and FA contributed equally to conceptualization, data collection, analysis, and manuscript writing, thus sharing first co-authorship. CS and JR provided indispensable analysis and inputs on results and discussion. All authors read and approved the final version of this article.

## Conflict of Interest

The authors declare that the research was conducted in the absence of any commercial or financial relationships that could be construed as a potential conflict of interest.

## Footnotes

1 Participants have not always responded to all of the socio-demographic questions (as they were not obligatory). As a result, the valid number of participants vary for each socio-demographic information.

2 The term “victimizing” is used throughout the text to express the attitude that the overindebted are victims of the social-economic context.
