# Supplementary material for: Perceived Causes and Attitudes Regarding Overindebtedness and Their Effects on Public Agreement With Government Financial Aid
Source: Front Psychol. 2021 Jun 17;12:591765. doi: 10.3389/fpsyg.2021.591765 (PMC8248491; doi:10.3389/fpsyg.2021.591765)
Supplement: Supplementary file 1 [file Table_1.pdf]

Supplementary Material

Table 1. Descriptive analysis of the items of the measure of causal attributions of overindebtedness

Items	*M*	*SD*	*Sk*	Sk/SE_{Sk}	*Ku*	Ku/SE_{ku}
1. Increase of the household	2.93	1.44	-0.04	-0.31	-1.28	-4.65
2. Late salary payments	3.57	1.57	-0.71	-5.15	-1.08	-3.95
3. Unstable work conditions	3.41	1.52	-0.49	-3.51	-1.20	-4.30
4. Salary cuts	3.74	1.48	-0.90	-6.58	-0.66	-2.41
5. Unemployment	3.95	1.60	-1.15	-8.34	-0.45	-1.62
6. Unemployment of spouse	3.67	1.53	-0.85	-6.13	-0.80	-2.89
7. Divorce/separation	3.30	1.44	-0.44	-3.18	-1.11	-4.02
8. Disease/work incapacitation	3.80	1.46	-0.99	-7.26	-0.48	-1.75
9. Impulsive buying	3.57	1.46	-0.72	-5.18	-0.88	-3.20
10. Bad management of monthly budget	3.78	1.30	-0.93	-6.72	-0.21	-0.78
11. Lack of appropriate knowledge about credit	3.60	1.33	-0.71	-5.20	-0.60	-2.18
12. Misfortune in financial issues	2.63	1.35	0.29	2.13	-1.08	-3.94
13. Problems with Guarantor	2.61	1.46	0.38	2.55	-1.20	-4.31
14. High taxes	3.28	1.38	-0.35	-2.50	-1.07	-3.92
15. Easy access to credit	3.65	1.32	-0.77	-5.63	-0.47	-1.71
16. Unsuccessful financial investments	3.25	1.48	-0.36	-2.56	-1.25	-4.47
17. Bad advice by banking entity	3.35	1.38	-0.46	-3.30	-0.98	-3.52
18. Failure in individual business ventures	3.37	1.50	-0.50	-3.63	-1.16	-4.21
19. Reduction in overtime payment	2.72	1.28	0.09	0.61	-1.06	-3.78
20. Excessive resort to credit (Credit cards. personal credit)	4.02	1.33	-1.32	-9.66	0.52	1.89
21. Victim of scam or fraud	3.09	1.59	-0.16	-1.15	-1.53	-5.51
22. Refusal of precarious work	2.49	1.35	0.36	2.56	-1.07	-3.83
23. Difficulty adjusting to the new economic reality	3.31	1.23	-0.27	-2.00	-0.78	-2.87
24. Current financial crisis	3.57	1.19	-0.45	-3.31	-0.64	-2.35
25. Debt "Snowball" effect	4.08	1.29	-1.36	-9.99	0.66	2.46

Note. *M* = Mean; *SD* = Standard deviation; *Sk* = Skewness; SE_{Sk} = Standard Error of Skewness; *Ku* = Kurtosis; SE_{ku} = Standard Error of Kurtosis.

Table 2. Descriptive analysis of the items of the measure of attitudes towards the overindebted

Items	*M*	*SD*	*Sk*	Sk/SE_{Sk}	*Ku*	Ku/SE_{ku}
1. Overindebted people have not had the same opportunities as other people	2.76	1.35	0.23	1.63	-1.05	-3.77
2. People are overindebted usually due to circumstances beyond their control	3.23	1.27	-0.11	-0.80	-1.02	-3.65
3. Society is responsible for helping overindebted people	2.82	1.31	0.13	0.91	-1.04	-3.70
4. If overindebted people worked harder. they could escape their debt situation	3.28	1.25	-0.24	-1.73	-0.83	-2.96
5. Overindebted people are discriminated against.	3.02	1.29	-0.02	-0.15	-1.00	-3.57
6. Unemployed overindebted people could find jobs if they tried harder.	2.98	1.28	0.05	0.38	-0.95	-3.40
7. Overindebted people should not be blamed for their misfortune.	2.50	1.31	0.51	3.64	-0.85	-3.04
8. Overindebted people are less capable. in general compared to other people.	2.13	1.21	0.85	6.08	-0.25	-0.91
9. Overindebted people have a different set of values to other people.	2.17	1.14	0.64	4.54	-0.50	-1.80
10. Overindebted situations are due to overindebted people's irresponsible spending.	3.10	1.35	-0.07	-0.49	-1.10	-3.95

Note. *M* = Mean; *SD* = Standard deviation; *Sk* = Skewness; SE_{Sk} = Standard Error of Skewness; *Ku* = Kurtosis; SE_{ku} = Standard Error of Kurtosis.

Table 3. Means and standard deviations of responses to each factor in the measure of causal attribution from overindebted and non-overindebted participants in Study 1.

	Overindebted		Non-overindebted	
Causes of overindebtedness	*M*	*SD*	*M*	*SD*
Situational	3.44	1.24	3.89	0.74
Dispositional	3.76	1.01	4.05	0.89

Table 4. Means and standard deviations for attitudes in the measure of attitudes towards the overindebted in Study 2.

Attitudes towards the overindebted	*M*	*SD*
Victimizing	2.50	0.69
Blaming	3.34	0.78
